# Career choices matter: educating medical students on a diversity of practice settings

**Published:** 2019-03-13

**Authors:** Nicholas Sequeira, Flora Jung

**Affiliations:** 1 Faculty of Medicine, University of Toronto, Ontario, Canada

Throughout medical school and residency, learners encounter a series of decision points which have a significant influence on their future careers. While some distinctions are explored thoroughly through the curriculum and made quite evident, decisions regarding future practice setting are less intuitive for trainees: Urban or rural? Community or academic? Given that many medical schools are located in large cities, learning about rural or community practice without actively seeking out opportunities can be challenging. In order to bridge this knowledge gap, students at the University of Toronto organized a career development event targeted at pre-clerkship students. This event featured a panel of physicians who compared their different practice settings and the associated logistics, rewards, and challenges.

The panel consisted of four physicians from four different settings and was gender-balanced: a large academic center, a community hospital, an inner-city clinic, and a rural community. The panel was made up of two family physicians, one hematologist, and one rheumatologist. Moderators posed a series of student-submitted questions covering various topics including day-to-day lifestyle, opportunities for teaching and research, and compensation differences between settings. After the 45-minute panel and 10-minute Q&A, students had the opportunity to connect one-on-one with the panelists.

Following the panel, 53 students (61% of attendees) completed an optional survey asking them to rate their agreement with panel objectives on a 5-point Likert scale and to provide qualitative feedback. While the event was open to all medical students, all survey participants were in their 1^st^ or 2^nd^ year of medical school. A total of 84.9% of respondents agreed or strongly agreed that they gained new knowledge about different practice settings, while 88.7% agreed or strongly agreed that the event was a valuable supplement to their formal medical education. In examining the qualitative feedback, the two most common themes were that the students appreciated the “good mix of practices/physician perspectives” and that they felt the panelists provided “very honest and transparent answers.” Of the 36 individuals who provided an answer for “what they liked most” about the panel, 17 mentioned the diversity of perspectives presented and 9 mentioned the candor of the speakers. In fact, 8/25 students who responded to “what they liked least” mentioned that they wanted even more panelists representing an even greater diversity of settings and specialties.

Survey results indicate that a substantial majority of medical students acquired new information from the panel and felt it was a valuable addition to their formal education. As such, the event may have value as a co-curricular activity intended to promote career exploration. Next steps include expanding the panel to involve more specialties and settings, increasing the number of items in the post-event survey, and performing a follow-up evaluation regarding elective choices to elucidate any long-term effects of attending the event. Moving forward, we will use our survey results to improve future iterations of the panel at the University of Toronto and hope that our results will inform similar initiatives at medical schools across Canada.

